# The Influence of Lignin Derivatives on the Thermal Properties and Flammability of PLA+PET Blends

**DOI:** 10.3390/ma18174181

**Published:** 2025-09-05

**Authors:** Tomasz M. Majka, Rana Al Nakib, Yusuf Z. Menceloglu, Krzysztof Pielichowski

**Affiliations:** 1 Cracow University of Technology, Faculty of Chemical Engineering and Technology, Department of Chemistry and Technology of Polymers, ul. Warszawska 24, 31-155 Kraków, Poland; 2 Cracow University of Technology, Interdisciplinary Center for Circular Economy, ul. Warszawska 24, 31-155 Kraków, Poland; 3 Sabanci University, Faculty of Engineering and Natural Sciences, Üniversite Caddesi No: 27, 34956 İstanbul, Tuzla, Turkey

**Keywords:** biopolymer, bio-blend, biocomposite, polylactide, poly (ethylene terephthalate), flammability, thermal properties, pyrolysis, circular economy

## Abstract

This paper presents a detailed analysis of the thermal and flammability properties of polylactide- (PLA) and poly(ethylene terephthalate)- (PET) based polymer blends with biofillers, such as calcium lignosulfonate (CLS), lignosulfonamide (SA) and lignosulfonate modified with tannic acid (BMT) and gallic acid (BMG). Calorimetric studies revealed the presence of two glass transitions, one cold crystallization temperature, and two melting points, confirming the partial immiscibility of the PLA and PET phases. The additives had different effects on the temperatures and ranges of phase transformations—BMT restricted PLA chain mobility, while CLS acted as a nucleating agent that promoted crystallization. Thermogravimetric analyses (TGA) analyses showed that the additives significantly affected the thermal stability under oxidizing conditions, some (e.g., BMG) lowered the onset degradation temperature, while the others (BMT, SA) increased the residual char content. The additives also altered combustion behavior; particularly BMG that most effectively reduced flammability, promoted char formation, and extended combustion time. CLS reduced PET flammability more effectively than PLA, especially at higher PET content (e.g., 65% reduction in PET for 2:1/CLS). SA inhibited only PLA combustion, with strong effects at higher PLA content (up to 76% reduction for 2:1/SA). BMT mainly reduced PET flammability (48% reduction in 1:1/BMT), while BMG inhibited PET more strongly at lower PET content (76% reduction for 2:1/BMG). The effect of each additive also depended on the PLA:PET ratio in the blend. FTIR analysis of the char residues revealed functional groups associated with decomposition products of carboxylic acids and aromatic esters. Ultimately, only blends containing BMT and BMG met the requirements for flammability class FV-1, while SA met FV-2 classification. BMG was the most effective additive, offering enhanced thermal stability, ignition delay, and durable char formation, making it a promising bio- based flame retardant for sustainable polyester materials.

## 1. Introduction

In the face of the growing climate crisis and the need to reduce fossil fuel consumption and greenhouse gas emissions, sustainable development technologies and strategies are evolving rapidly. The concept of the Circular Economy (CE) has gained particular importance, aiming to maximize the use of raw materials through reuse, recycling, and designing products for reprocessing [1,2,3,4]. One of the key challenges of the CE remains the development of advanced polymer materials that meet both functional and environmental requirements, including mechanical strength, thermal stability, biodegradability, and fire safety [5,6,7,8].

Polylactide (PLA), a biodegradable polyester derived from renewable raw materials (primarily corn starch or sugarcane), has garnered significant interest in recent years as a potential alternative to conventional plastics, such as polyolefins [9,10]. Its main advantages are biodegradability, renewable origin, good processability, and transparency [11,12]. However, pure PLA lacks several properties required for broad engineering applications, most notably it shows low thermal resistance, brittleness, and high flammability [13,14,15,16,17]. Typical pristine PLA exhibits a limiting oxygen index (LOI) of approximately 19% (range 19–21%), and a peak heat release rate (PHRR) often exceeding 385 kW/m^2^, underscoring its inherent high flammability [18].

To improve the functional properties of PLA, it is often combined with other polymers in the form of blends [19,20,21], one of the most frequently studied being poly (ethylene terephthalate) (PET)—a polymer with very good mechanical and thermal strength, but which is non-biodegradable material [22]. Although PET and PLA are thermodynamically immiscible [19], their blends make it possible to obtain after compatibilization materials with a balanced combination of properties; however, their high flammability and limited thermal stability (significantly reduced Heat Deflection Temperature (~8 K), limited thermal integrity by active phase separation and dual T_g_ and much lower decomposition onset (~80 K)) remain problematic, especially in the context of fire safety that is of primary importance in, e.g., aerospace and electrical industries [23,24,25].

For this reason, research into the use of flame retardants, i.e., additives that reduce the flammability of polymer materials, is becoming increasingly important. Classic flame retardants, such as halogenated or phosphorus compounds, although effective, raise numerous environmental and health concerns—their use is associated with the emission of toxic gases during combustion, accumulation in the environment, and difficulties in recycling [26,27,28]. A promising solution to these problems is the use of flame retardants of natural origin, based on biopolymers, biomass, or plant compounds. These constitute an attractive alternative to conventional flame retardants due to their biodegradability, non-toxicity, and potential compatibility with the CE [29,30,31,32].

Among natural flame retardants, lignin, phytic acid, tannins, chitin, and modified biomass phosphates are particularly notable, as they can effectively reduce the release of heat and smoke during combustion without impairing the mechanical properties of the material [33,34,35,36,37]. Lignin and its sulfonate derivatives, which are waste products from pulp and paper industry, exhibit natural thermal resistance and the ability to form charred protective layers due to their aromatic structure [38,39]. In turn, phytic acid (a compound present in cereal seeds) contains six phosphate groups, making it an effective source of phosphorus in the mechanisms that slow down combustion [40,41,42,43]. For lignin-based systems, adding ~15 wt.% modified lignin can reduce peak heat release rate by over 60% in polypropylene matrices, demonstrating significant char-forming capacity [44]. Similarly, phytic acid–based flame retardants have delivered up to 57% reductions in total heat release and 80% in total smoke release in treated wood composites [40].

In the context of the CE, it is important not only to use renewable raw materials but also to effectively manage waste streams. The use of natural flame retardants obtained from plant or agro-industrial waste (e.g., sewage sludge, agricultural waste, wood residues) fits perfectly into this paradigm. Such solutions enable simultaneous reduction in material flammability, improvement of operational safety, and closure of the product life cycle in the spirit of the circular economy. A life cycle assessment that compared PLA panels with natural fiber reinforcement and non-halogenated flame retardants to conventional panels found a CO_2_-equivalent savings of up to 6000 kg per square meter during use phase [45]. LCA studies of PLA biocomposites demonstrate significantly lower environmental impact than fossil-based plastics, although end-of-life impacts remain a minor fraction compared to manufacturing and use phases [46].

In previous publications, we investigated the use of calcium lignosulfonate (CLS), lignosulfonamides (SA) synthesized from CLS, and CLS modified with tannic acid (TA), as potential additives reducing the flammability of PLA. In [47], the quantitative and qualitative influence of the form of natural phenolic compounds (NPC) on the degradation of PLA was examined under various measurement conditions. The results of these analyses showed that TA is neither an effective flame retardant nor a highly swelling material; however, in combination with CLS, it can produce an interesting synergistic effect. For example, the physical mixture of TA + CLS improves the thermal stability of PLA during processing. Whereas the chemically obtained hybrid material BMT allows for a 30% reduction in flammability compared to PLA, which cannot be achieved by physically mixing these components in a polymer melt. In [48] it was shown that lignosulfonamides reduce PLA flammability by up to 40%. The lowest flammability was obtained at 9 wt.% SA, and this effect was attributed to the release of SO_2_ at an early stage of biocomposite decomposition. This favored the simultaneous formation of coke and char, which acted as a local insulator.

This publication aims to comprehensively present the results of research on the influence of selected natural flame retardants on the flammability and structural properties of PLA+PET blends. In particular, it analyzes changes in the flash point, heat release, and thermal degradation mechanisms, as well as the compatibility of these additives with the polymer matrix. Potential recycling and biodegradation pathways for such blends are also examined, which is an important step toward implementing sustainable material solutions.

The use of an interdisciplinary approach enables a reliable assessment of the tested solutions as viable alternatives to conventional polymer materials currently used in industry. The primary goal is to identify directions for the development of materials aligned with the principles of the circular economy, materials that not only reduce environment impact but also meet the growing functional demands of advanced applications.

## 2. Materials

Polylactide (PLA, CAS No. 26100-51-6) with the trade name Ingeo™ Biopolymer 3052D was purchased from NatureWorks (Blur, Plymouth, MN, USA).

Poly (ethylene terephthalate) (PET, CAS No. 25038-59-9) with the trade name NEOPET80 was acquired from NeoGroup (Vilnius, Lithuania).

Calcium lignosulfonate (CLS, CAS No. 8061-52-7) with the trade name Bretax C (BX) was purchased from Mosaico S.p.A. (Tolmezzo, Italy).

Didodecyl-lignosulfonamide (SA) was obtained according to the procedure described in [48,49], where CLS was subjected to a two-step reaction first with PCl_5_ (CAS No. 10026-13-8, Sigma Aldrich, Darmstadt, Germany) to obtain lignosulfonyl chloride, which in the second stage reacted with secondary amine–didodecylamine (CAS No. 3007-31-6, Sigma Aldrich, Darmstadt, Germany).

CLS modified with tannic acid (BMT) was prepared according to the procedure described in [47], where tannic acid (TA, CAS No. 1401-55-4) provided by Sigma Aldrich (Darmstadt, Germany), was appropriately adsorbed on the CLS surface.

CLS modified with gallic acid (BMG) was obtained in a similar procedure as BMT, where 100 g of CLS, previously dried at 363 K for 24 h under vacuum, was placed in a round-bottom flask and 700 mL of ethylene glycol (CAS No. 107-21-1, Chempur, Piekary Śląskie, Poland) was introduced into the vessel. The mixture was heated to 338 K, and 100 g of gallic acid (CAS No. 149-91-7, Sigma Aldrich, Darmstadt, Germany) was added. The mixture thus prepared was brought to 363 K, and this condition was maintained for 5 h. After cooling to room temperature, the mixture was concentrated on a rotary evaporator at 338 K, at 50 rpm, at a pressure of 500 mbar, until the solvent was completely evaporated. The resulting precipitate was pre-dried to a constant weight at 353 K for 168 h, and then at room temperature for 24 h. The final product (BMG) was obtained as a viscous paste of 130 g (65% yield).

## 3. Sample Preparation

**Figure 1:**
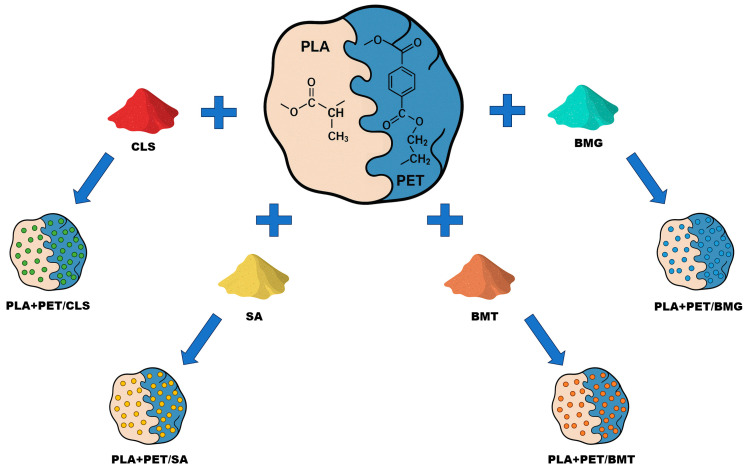
The diagram showing sample preparation process of PLA+PET composite blends.

In the preparation process of PLA+PET composite blends (Figure 1), a processing line consisting of a Brabender DR20 feeder (RHL-Service, Poznań, Poland), twin screw extruder Haake Rheomex OS PTW 16/25 (RHL-Service, Poznań, Poland), cooling bath Zamak W1500 (Zamak Mercator, Skawina, Poland) and pelletizer Zamak G–16/325 (Zamak Mercator, Skawina, Poland) was used.

Using this processing line, PLA+PET/CLS, PLA+PET/SA, PLA+PET/BMT, and PLA+PET/BMG composite blends containing 3 wt.% of filler, were obtained. Also in the polymer blends, three different mass ratios of PLA to PET were used: 1:2, 1:1, 2:1. Standard samples for LOI and UL-94 testing were obtained using a P-200 press (Zamak Mercator, Skawina, Poland). The processing conditions have been shown in Table 1.

Table 2 presents a summary of the obtained composite blends along with the designations used in the work.

## 4. Methods

Differential scanning calorimetry (DSC) and thermogravimetry (TG) techniques were used for thermal analysis.

The biocomposite blends were characterized using a Mettler Toledo DSC823e apparatus (Mettler-Toledo, Greifensee, Switzerland). The test was performed in a nitrogen atmosphere according to the following temperature programs:

(1) heating 298–553 K at a rate of 10 K/min,

(2) cooling 553–273 K at a rate of 10 K/min,

(3) heating 273–553 K at a rate of 10 K/min.

TGA was performed using a Netzsch TG 209F1 Libra apparatus (Netzsch Group, Selb, Germany) in both oxidizing and inert atmospheres. The test was carried out under the following conditions: a temperature range of 303 to 873 K and a heating rate of 10 K/min.

Flammability tests included Micro Combustion Calorimetry (MCC), Vertical (VB) and Horizontal (HB) UL-94 flammability tests, and Limited Oxygen Index (LOI). These tests were complemented by Fourier Transform InfraRed (FTIR) spectroscopy.

MCC was performed using the Fire Testing Technology apparatus (FTT, East Grinstead, Great Britain) according to ASTM D7309. Composite tests were performed in the temperature range of 303–1023 K at a heating rate of 1 K/s.

UL-94 VB and HB flammability tests were conducted on bar-shaped samples by IEC 60695-11-10:1999/AMD 1:2003. Total burning time and burning rate were measured, unless the material exhibited a self-extinguishing effect under the specified test conditions. Digital photographs of the burned samples and residues were taken using a VEVOR JD-109 thermal camera (London, UK).

LOI was measured according to DIN EN ISO 4589-2 using a Concept Fire Testing Oxygen Index Module apparatus (FTT, East Grinstead, Great Britain). The oxygen concentration in the gas stream was adjustable with a volumetric accuracy of 0.1%. The flow rate of the nitrogen/oxygen mixture was maintained at 10 L/min.

In order to explain the combustion mechanism of composite blends, FTIR measurements were performed on both pre-combustion and post-combustion samples. A PerkinElmer Spectrum 65 spectrometer (PerkinElmer Inc., Waltham, MA, USA) equipped with an ATR attachment and a diamond/ZnSe crystal was used for the FTIR analysis. The tests were performed using the spectral range of 4000 to 400 cm^−1^ with 32 scans.

The morphological investigations were carried out by a Zeiss LEO Supra 35VP SEMFEG Scanning Electron Microscope (Carl Zeiss AG, Oberkochen, Germany). The SEM images were acquired at an accelerating voltage of 5 kV and a 10 mm working distance. Prior to imaging, each sample was coated with a 4 nm thick layer of gold.

## 5. Results and Discussion

### 5.1. Analysis of Biofillers

**Figure 2:**
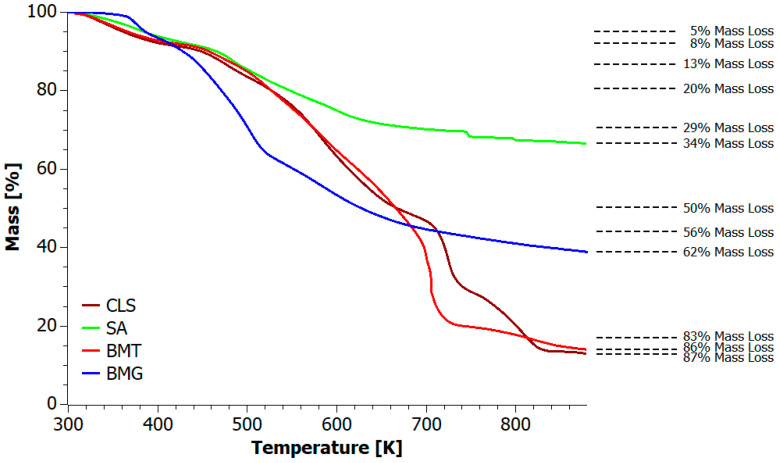
The thermogravimetric curves of the biofillers: CLS, SA, BMT, and BMG.

Four biofillers were used to prepare composite blends: CLS, SA, BMT, and BMG. The first step involved analyzing their thermal properties, particularly their decomposition behavior under oxidizing conditions and during microcalorimetric combustion. Figure 2 displays the thermogravimetric analysis (TGA) curves of the biofillers, and Table 3 summarizes the corresponding TGA indices. The data indicate that approximately 16 wt.% of CLS, SA, and BMT, and 34 wt.% of BMG decompose at the PLA processing temperature.

CLS consists of various phenylpropanoid units, such as coniferyl, p-coumaryl and sinapyl alcohols, which are rich in active hydrophilic functional groups, including sulfonic acid, aromatic and aliphatic hydroxyl groups, as well as hydrophobic alkyl chains [50,51]. Upon depolymerization, at temperatures above 423 K, CLS yields five main groups of low-molecular-weight compounds, including phenolics, guaiacol and syringol derivatives, and esters [52]. The decomposition of CLS occurs in three main phases: (i) 303–423 K, characterized by the loss of physically bound water, (ii) 423–673 K, marked by the emission of CO, CO_2_, SO_2_, mercaptans, and dehydration water, and (iii) above 673 K, involving condensation and breakdown of phenolic intermediates [53]. TG analysis revealed that unmodified CLS exhibited the lowest thermal stability among all the tested biofillers, with the onset of water loss observed as early as 361 K. The decomposition followed a complex six-stage mechanism, with the most significant mass loss occurring during the fifth stage (687–751 K). This phase is likely associated with intense oxidation, hydroxymethylation, and sulfomethylation reactions of phenolic compounds. Adjacent stages, spanning over 499–681 K and 757–848 K, may involve emission of non-flammable volatile gases such as CO_2_, CO, and SO_2_, which contributes to the minimal char residue observed at 873 K.

SA exhibited the highest thermal stability among all tested materials. During thermo-oxidative decomposition, SA did not reach the T_50%_ point and retained the highest residue after decomposition. The thermal decomposition was a multi-stage process (over six stages) and complex. It included a very short water release stage up to 310 K, emissions of highly volatile gases up to 437 K, and a wide decomposition range from 437–736 K associated with formation of CO, CO_2_, hydrocarbons, nitrogen oxides, and amine vapors, which then decomposed successively at 747 K, 764 K and 798 K [54].

Both tannic acid (TA) and gallic acid (GA) are hydrolysable tannins. GA is the basic building block of gallic tannins and can form ester bonds with sugars (mainly glucose), creating complex structures that are easily hydrolyzed. TA, on the other hand, contains a central polyhydric alcohol core linked to gallic acid by ester bonds [55]. This structural difference led to distinct decomposition mechanisms for BMT and BMG. BMT decomposed in three stages—the first stage (307 K) involved the rapid evaporation of water bound to CLS. The second, longer stage was associated with the gradual release of acetic acid, 1,2,3-benzenetriol, and carbon dioxide, due to the breakdown of the outer layer of gallic acid units, with maximum degradation near 700 K. The final stage (701–743 K) was characterized by the rapid emission of large amounts of CO and CO_2_ [56,57,58]. Overall, TA did not significantly improve the thermal stability of BMT compared to CLS, with performance remaining similar in certain temperature ranges.

BMG decomposed in four distinct stages. The first stage in the range of 302–397 K included both the evaporation of water and the cleavage of the GA backbone, accompanied by the loss of hydroxyl groups [59,60]. The second and most significant stage occurred between 403–539 K where gallic acid underwent decarboxylation yielding pyrogallol and a range of organic acids such as vanillic acid, phthalic acid, benzoic acid, ellagic acid, and residual tannic acid [61,62]. Several of these decomposition compounds, including vanillic acid [63,64,65,66], phthalic acid and benzoic acid [67,68], as well as ellagic acid [69,70], are reported in the literature to possess flame retardant properties. The third stage at 546–692 K corresponded to the breakdown of these compounds into aromatic hydrocarbons, phenolic compounds, CO_2_, CO, along with the formation of char. The final stage, above 692 K, was mainly associated with the slow oxidative combustion of the resulting char residue.

**Figure 3:**
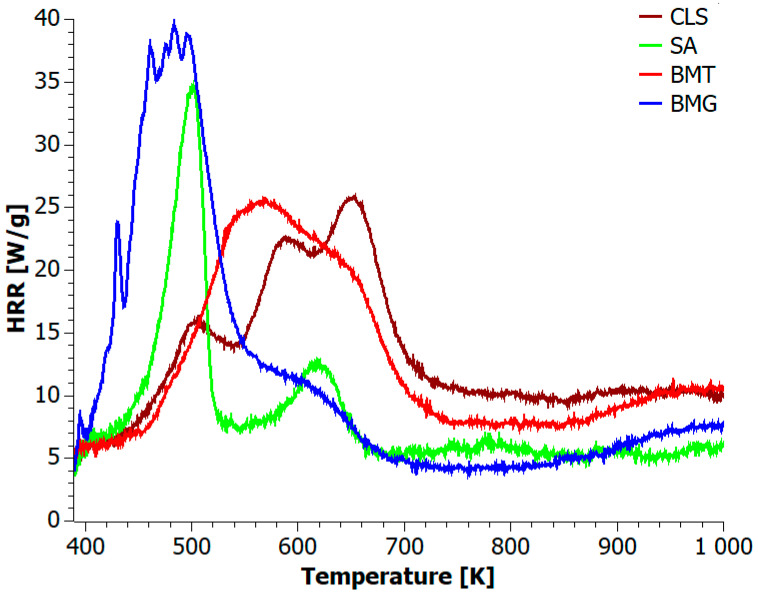
HRR vs. temperature profiles for biofillers.

The results of the Heat Release Rate (HRR) measurement as a function of temperature are shown in Figure 3. Among the tested biofillers, BMT exhibited the shortest burning duration (271 s), while SA showed the longest (443 s). The lowest PHRR was observed for CLS and the highest for BMG (see Table 4). Notably, none of the samples exceeded a PHRR of 40 W/g, indicating their potential classification as low-flammable biomaterials. It is important to highlight that during microcalorimetry combustion, the heating rate was approximately six times higher than that used in thermogravimetric analysis, which may have influenced the thermal degradation pathways by promoting faster devolatilization and altering char formation route.

In general, all samples burned in three stages, with the first combustion stage dominating in the SA, BMT, and BMG biofillers, and the third stage dominating in the CLS material. This suggests that an increased concentration of polyphenols contributes to their decomposition into volatile flammable gases during the first phase of combustion, leading to an earlier onset of char formation. For example, the HRR curve for CLS showed three peaks at 505, 587, and 653 K. These peaks correspond to the decomposition of phenolic compounds through intermediate compounds, such as mercaptans to CO, CO_2_, and SO_2_. Noteworthy, no char combustion was observed above 700 K.

The first combustion phase of didodecylsulfonamide was rapid, exhibiting a sharp maximum at 500 K. This phase is associated with the release of aliphatic hydrocarbons and polyphenols from the sulfonamide group and their decomposition into high-energy volatile products. The second stage at 616 K can be attributed to the phase transformations of the sulfonamide residue and its decomposition into CO, CO_2_, SO_2_, and char. The third stage, observed as a broad halo with a maximum at 776 K, was associated with the oxidative combustion of the formed char [71,72,73].

In the work [74], tannic acid was found to be an excellent natural flame retardant. To initially assess this, the heat emission curve during the combustion of BMT was analyzed. The initial analysis shows that the first two combustion stages overlap, forming a broad peak between 468 and 745 K with a maximum at 569 K. This result suggests that tannic acid delays the release of polyphenols present in CLS, as it first decomposes at temperatures up to 633 K into 1,2,3-benzenetriol and carbon dioxide. Above the PHRR point, the cross-linked structures of tannic acid and sulfonamide residues oxidize to CO and CO_2_, resulting in a low char yield under oxidizing conditions [75,76,77].

A very similar trend was observed for the BMG material. However, in this case, gallic acid adversely affects CLS. First, the sample ignites after 3 s at a relatively low temperature of 382 K. Second, HRR curve analysis shows that the combustion mechanism of BMG during the first phase was complex, with its peak occurring much earlier than in CLS and BMT. According to the literature, CO_2_ is the main component of exhaust gases during both the first and second decomposition phases, although some authors also report the formation of water vapor [78]. The formation of CO_2_ is mainly related to the decarboxylation of gallic acid [79]. The third decomposition stage occurs above 865 K, similarly to BMT combustion. This means that the char formed in both cases remains rich in flammable compounds and tends to glow and ignite unexpectedly at high temperatures.

### 5.2. Analysis of Composite Blends

**Figure 4:**
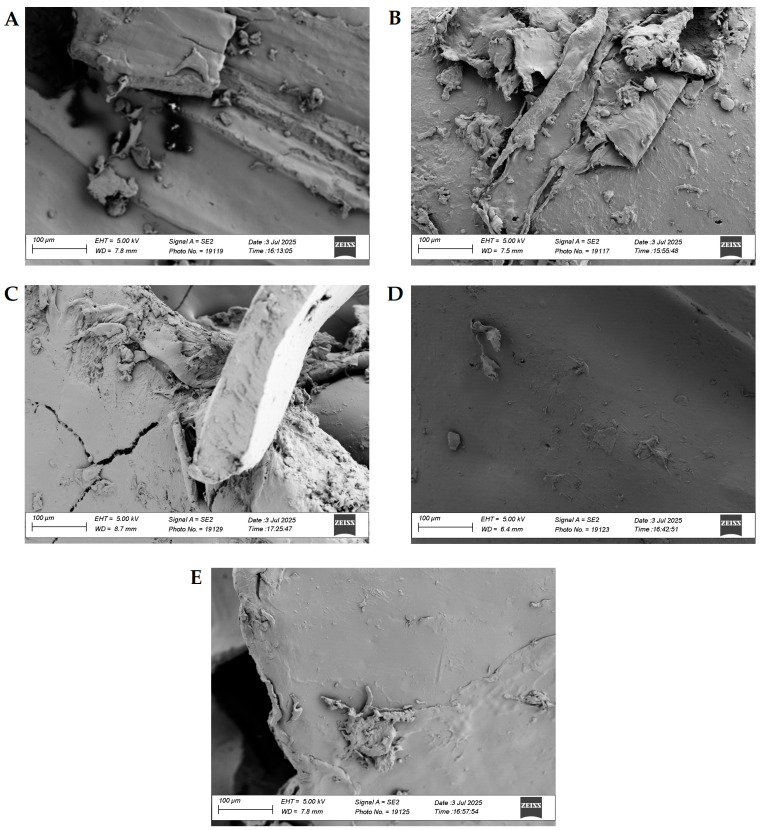
SEM images of PLA+PET composite blends, Where (**A**) 1:1; (**B**) 1:1/CLS; (**C**) 1:1/SA; (**D**) 1:1/BMT; (**E**) 1:1/BMG.

Figure 4 presents SEM images of selected 1:1 composite blend samples at 500× magnification. Figure 4A shows a heterogeneous surface with distinct lamellar fragments and agglomerates. Interfacial cracks appeared, suggesting poor adhesion between the PLA and PET phases in this region. The structure indicated partial phase separation, with visible voids and defects. Therefore, the morphology of the 1:1 sample indicated limited compatibility, where the phases were not fully integrated. It was concluded that the presence of voids and phase boundaries may promote local overheating and early decomposition (primarily due to moisture trapped between the phases) [80,81]. Furthermore, delamination may facilitate oxygen diffusion and the escape of volatile products, which can shorten the time to ignition and increase the combustion rate (increased tendency for melt dripping).

In turn, the addition of CLS (Figure 4B) made the blend surface rougher and porous, with visible fragments of detached layers. Clear traces of delamination were observed, suggesting weak interfacial bonds. This fibrous structure may indicate the elongation of one of the phases during the deformation process and the presence of larger PET or PLA domains. Furthermore, the observed delamination may create thermal conduction paths and cracks initiating degradation [82,83]. The layered structure of the 1:1/CLS sample, on the other hand, may indicate a large active surface area, which is expected to result in higher PHRR and more intense smoke.

Even the introduction of lignosulfonamide (Figure 4C) did not result in good mixing at the molecular level. The two coexisting phases could be identified by the nature of the surface, where the PET phase was smooth and compact, and the other PLA phase was weakly bonded. As in the previous samples, cracks were visible in the structure, suggesting a lack of full coalescence of the components. The structure of the 1:1/SA blend was the weakest of all the composite blends analyzed. It was concluded that the interfaces present within this sample may constitute starting points for thermo-oxidative degradation. This could also result in rapid sample ignition, rapid emission of volatile products, and a high risk of dripping burning material.

A significant change in the morphology of the PLA+PET samples occurred after the BMT incorporation into the system (Figure 4D). The surface of the analyzed sample was relatively smooth and uniform, although only a few minor inclusions were visible. No visible cracks, crevices, or large phase separations were observed, indicating better phase dispersion and possibly higher compatibility. The observed higher degree of homogeneity may contribute to an increased degree of crystallinity, which in turn may reduce the diffusion of heat and volatile products [84,85,86,87]. Based on this analysis, a higher onset temperature of mass loss, a longer time to ignition, a lower value of PHRR coefficient, and a reduced tendency to drip were expected.

A structure very similar to 1:1/BMT was obtained after the addition of BMG to the blend (Figure 4E). The observed surface was generally smooth, but small clusters and agglomerates appeared, although they were not as large and distinct as in Figure 4A–C. This may suggest that local defects can initiate thermal decomposition and serve as a point of ignition for the sample, although the overall homogeneity should improve flame resistance.

**Figure 5:**
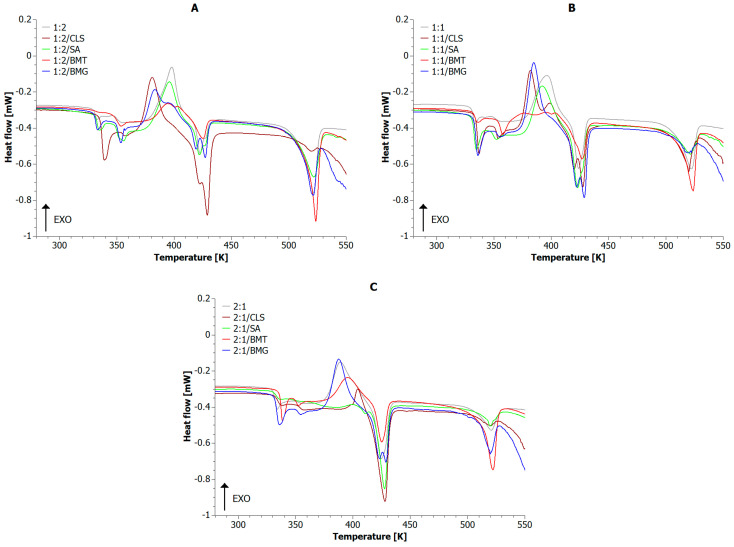
DSC curves for composite blends where the PLA:PET ratio is (**A**) 1:2; (**B**) 1:1; (**C**) 2:1.

Figure 5 shows the DSC plot for the PLA+PET composite blends and reference samples. Based on graphical interpretation, DSC indicators were determined and are presented in Table 5. The degree of crystallinity was calculated using Equation (1) according to [88,89,90,91,92,93]:

(1) $$X_{C}=\left[\gamma_{PLA}\cdot \left(\frac{{∆H}_{m\_PLA}-{∆H}_{CC\_PLA}}{\left(1-\omega \right)\cdot {∆H}_{PLA}^{{}^{\circ}}}\right)\right]+\left[\gamma_{PET}\cdot \left(\frac{{∆H}_{m\_PET}-{∆H}_{CC\_PET}}{\left(1-\omega \right)\cdot {∆H}_{PET}^{{}^{\circ}}}\right)\right]$$

where:

${∆H}_{m\_PLA}$—melting enthalpy of a PLA’s sample.

${∆H}_{m\_PET}$—melting enthalpy of a PET’s sample.

${∆H}_{CC\_PLA}$—enthalpy of cold crystallization for PLA’s sample.

${∆H}_{CC\_PET}$—enthalpy of cold crystallization for PET’s sample.

${∆H}_{PLA}^{{}^{\circ}}$—melting enthalpy of a completely crystalline polylactide equal to 93 J/g [94].

${∆H}_{PET}^{{}^{\circ}}$—melting enthalpy of a completely crystalline poly (ethylene terephthalate) equal to 135 J/g [95].

$\gamma_{PLA}$—share of PLA phase in the blend.

$\gamma_{PET}$—share of PET phase in the blend.

ω—filler content.

**Table 5:** DSC indicators Where PLA phase melting temperature (T_m_PLA_), PET phase melting temperature (T_m_PET_), PLA phase melting enthalpy (ΔH_m_PLA_), PET phase melting enthalpy (ΔH_m_PET_), total degree of crystallinity (X_c_), PLA phase degree of crystallinity (X_c_PLA_), PET phase degree of crystallinity (X_c_PET_), cold crystallization temperature (T_cc_), PLA phase glass transition temperature (T_g_PLA_), PET phase glass transition temperature (T_g_PET_).

Sample	T_g_PLA_ [K]	T_g_PET_ [K]	T_cc_ [K]	T_m_PLA_ [K]	T_m_PET_ [K]	∆H_m_PLA_ [J/g]	∆H_m_PET_ [J/g]	X_c_PLA_ [%]	X_c_PET_ [%]	X_c_ [%]
1:2	330.19	350.55	397.97	422.54	521.79	8.92	28.12	13.10	27.44	40.55
1:2/CLS	335.81	355.25	380.79	428.95	519.33	31.08	1.89	20.87	14.48	35.35
1:2/SA	331.80	350.08	395.62	422.19	521.61	9.41	22.65	12.77	24.37	37.14
1:2/BMT	331.60	350.43	394.27	425.22	523.57	4.97	32.94	6.13	22.49	28.62
1:2/BMG	330.45	351.24	383.11	427.17	521.39	11.55	18.21	12.80	21.14	33.94
1:1	330.97	351.55	396.13	423.84	522.29	14.07	20.01	22.33	17.59	39.92
1:1/CLS	333.88	352.41	381.97	427.65	519.96	21.48	12.85	28.68	16.51	45.19
1:1/SA	331.34	349.41	391.95	423.15	521.32	18.58	10.16	25.32	14.28	39.60
1:1/BMT	332.61	355.58	---	426.84	523.74	16.48	22.06	9.00	8.29	17.29
1:1/BMG	332.73	353.07	384.81	428.8	518.82	26.89	4.52	17.37	3.56	20.93
2:1	331.41	352.08	388.95	424.81	520.48	19.04	11.06	32.18	9.13	41.31
2:1/CLS	333.71	354.12	404.10	427.97	519.19	31.81	2.25	26.60	1.77	28.37
2:1/SA	332.59	368.55	399.25	427.65	519.52	24.38	5.67	18.23	1.60	19.82
2:1/BMT	335.57	350.26	273.15	395.43	425.18	11.45	23.46	19.44	9.60	29.04
2:1/BMG	332.29	351.58	387.62	428.65	519.79	22.18	9.17	34.22	8.45	42.67

During the calorimetric measurement in the temperature range of 273–553 K, the following phase transformations occurred: 320–360 K—glass transition, 360–410 K—cold crystallization, 410–550 K—melting and thermal degradation of the blends. In all the analyzed samples, three main observations were noted:

Two distinct glass transition temperatures, suggesting partial immiscibility of the PLA and PET phases, i.e., at least a two-phase polymer system.

One cold crystallization temperature, assigned mainly to the PLA phase, indicating that the PET phase was already partially crystallized in all cases.

Two melting temperatures characteristic of both polymer phases (390–430 K for PLA and 515–530 K for PET).

**Figure 6:**
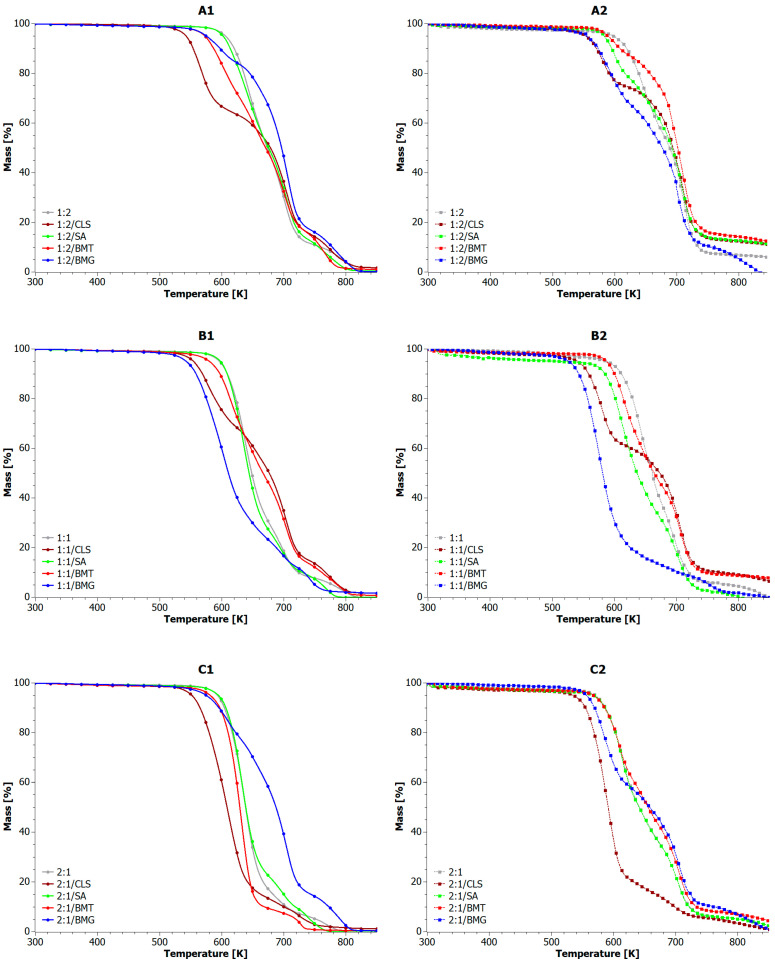
TGA curves for composite blends. Where (**A1**) the PLA:PET blends in ratio of 1:2 were investigated under oxygen conditions; (**B1**) the PLA:PET blends in ratio of 1:1 were investigated under oxygen conditions; (**C1**) the PLA:PET blends in ratio of 2:1 were investigated under oxygen conditions; (**A2**) the PLA:PET blends in ratio of 1:2 were investigated under inert atmosphere; (**B2**) the PLA:PET blends in ratio of 1:1 were investigated under inert atmosphere; (**C2**) the PLA:PET blends in ratio of 2:1 were investigated under inert atmosphere.

It is worth noting that, compared to the thermogravimetric analysis performed under an inert atmosphere (Figure 6(A2,B2,C2)), the mass loss at 550 K (using the same heating rate) did not exceed 5%.

When analyzing the T_g_ values recorded for all types of composite blends, it was noted that the addition of BMT particularly shifted the glass transition temperature of the PLA phase toward higher temperatures. This suggests restricted mobility of polylactide chains and, de facto, stiffening of the biopolymer matrix. Similarly, the presence of CLS in all analyzed blend types caused a significant rightward shift in the glass transition temperatures of both polymer phases. The presence of SA and BMG additives in 1:2 and 1:1 blends had an insignificant effect on the T_g_ values. On the other hand, in the blend dominated by the PLA phase (2:1), the presence of SA caused a strong interphase shift, which may indicate significant amorphization of PET.

During the analysis of the second-phase transformation of 1:2 blends, it was found that the addition of CLS and BMG lowered the cold crystallization temperature (T_CC_). Thus, these compounds act as nucleating agents, facilitating primarily the crystallization of PLA. In blends with equal amounts of both polymer phases (1:1), the presence of CLS and BMG in the system facilitated PLA crystallization, while the addition of BMT completely suppressed this process, indicating an earlier crystallization of the biophase. Increasing the proportion of the biopolymer phase relative to the PET phase caused CLS to extend the PLA crystallization process, while BMT influenced the formation of artifacts exhibiting relatively low T_CC_ values.

The presence of two distinct melting phases in the reference samples 1:2, 1:1, and 2:1 suggests that there is no fusion of the polymer phases and that they are not fully compatible. This is confirmed by the stable differences between the melting points of T_m_PLA_ and T_m_PET_, which averaged 90 K for most of the tested samples. A possible fusion of the phases was observed only in the 2:1/BMT sample, where the difference between the two melting points was less than 30 K. In this case, the addition of BMT also lowered the melting temperatures of both phases, suggesting that the filler may disrupt the polymer structure or reduce its degree of order. In most cases, the additives had minor effect on the melting temperatures of both polymer phases. Generally, the presence of CLS and BMG caused a slight increase in the melting temperature of the PLA phase and a negligible decrease in the melting temperature of the PET phase across all blends.

In the reference blends 1:2, 1:1, 2:1, as well as in the composite blends, the mass fraction of the individual polymer phases generally corresponded to the degree of crystallinity of the respective phases. Only the addition of CLS to the 1:2 blend reversed this trend, favoring the biophase. Generally, CLS increased the degree of crystallinity of PLA, while reducing the value of X_C_PET_. It was also found that the addition of SA reduced the values of X_C_PET_. In all cases, BMT reduced the degree of crystallinity of both phases and thereby lowered the overall degree of crystallinity of PLA+PET blends.

An increase in the total degree of crystallinity improves the stiffness and barrier properties of composite blends. High X_C_ values usually mean more difficult ignition, slower emission of flammable gases, increased susceptibility to char formation, and reduced flame spread. Therefore, based on the DSC results, it can be predicted that 1:1/CLS and 2:1/BMG samples, characterized by a higher degree of crystallinity, should be more resistant to ignition, decompose slower, and form more char. Conversely, materials with lower X_C_, such as 1:1/BMT, may be more susceptible to ignition and decompose more rapidly, as they lack char protection.

Figure 6 shows the results of thermogravimetric analysis conducted under both oxidizing (1) and inert (2) conditions for comparison. Thermo-oxidative measurement of reference samples led to complete decomposition of blends to volatile gaseous products, while pyrolytic measurement led to the formation of char, the amount of which depended on the fraction of individual polymer phases.

Considering the thermo-oxidative degradation of the blends is of crucial importance, as the individual components exhibit decomposition mechanisms similar to those observed during intense combustion. The reference samples and those containing CLS, SA, and BMG decomposed in three stages, but the presence of some lignin derivatives favored the earlier initiation of individual decomposition stages. Among all the analyzed samples (under oxidative conditions, Table 6), a temperature range can be identified in which additives significantly affect thermal stability. This range occurs above 650 K, corresponding to mass loss exceeding 20%. Up to the T_20%_ point, the reference sample consistently exhibited the highest thermal stability, while the presence of additives promoted faster volatilization of at least 20 wt.% of the sample due to the release of low-molecular-weight gaseous products. Beyond the T_20%_ point, the presence of CLS, SA, and BMG in 1:2 blends, CLS, SA, and BMT in 1:1 blends, and BMG in 2:1 blends reversed the trend in favor of composite blends, which then exhibited higher thermal stability than the reference.

Among the 1:2 composite blends, the one with BMT addition exhibited the lowest thermal stability over the entire measurement range. The 1:2/BMT sample decomposed in four stages, where three consecutive stages were observed in the range of 530–740 K and the fourth stage in the range of 740–810 K. Similarly, the addition of CLS and BMG to 1:1 blends accelerated the onset of their degradation already at 480 K. Moreover, the 1:1/BMG sample was characterized by the main decomposition stage in the range of 480–670 K and two subsequent stages in the range of 670–790 K. Among the 1:1 blends, the BMG-containing sample demonstrated the lowest thermal stability in the entire measurement range. For the 2:1 blends, only the 2:1/BMT sample underwent a two-stage decomposition, where the main stage was observed in the 520–680 K range and the second one in the 700–740 K range.

Overall, the TG profile of the 1:2/SA, 1:1/SA, and 2:1/SA samples closely resembled those of their respective reference samples (1:2, 1:1, 2:1) across the entire temperature range. SA-containing blends exhibited the first two decomposition stages in the range of 540–740 K and the third in the range of 740–830 K. In general, the composite blends characterized by increased thermal stability, such as 1:2/BMG, 2:1/BMG, yielded small residue amounts post-decomposition. Conversely, samples with lower thermal stability produced greater amounts of char under oxidizing conditions.

For comparison, all composite blends tested under an inert atmosphere decomposed in two stages over a broad temperature range of 500–780 K, which is consistent with previous reports [96,97]. The thermal degradation proceeded more slowly than in oxidizing conditions, and the residue after decomposition was significantly larger, reaching up to 10.5% (see sample 1:2/BMT). Typically, samples containing BMT and SA showed higher residue and higher T_MAX_ values, indicating better resistance to decomposition in anaerobic conditions. On the other hand, the addition of CLS and BMG often led to a decrease in T_5%_ and T_MAX_ indices, which could result from their lower decomposition temperature, tendency to initiate the degradation process, and possible interactions with matrix. Table 7 compares the most important features of composite blend samples obtained under thermo-oxidative (OX) and pyrolytic (IN) degradation conditions.

**Figure 7:**
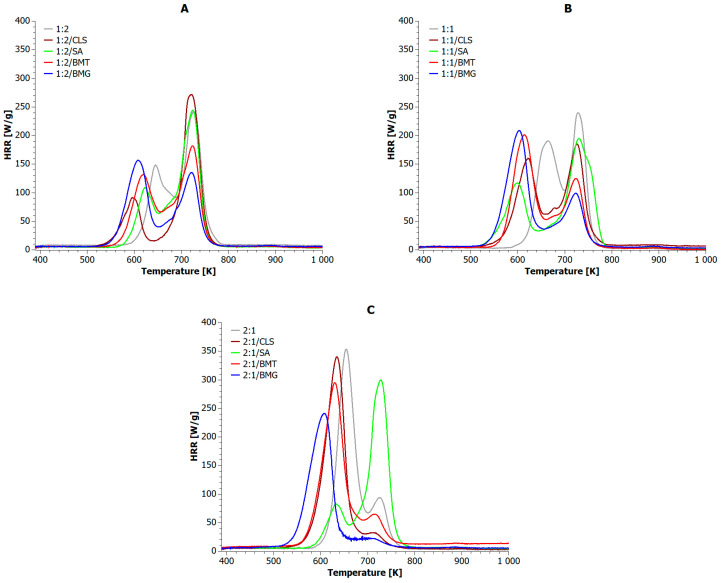
Heat release rate vs temperature for PLA+PET blend composites Where (**A**) the PLA:PET phases in ratio of 1:2; (**B**) the PLA:PET phases in ratio of 1:1; (**C**) the PLA:PET phases in ratio of 2:1.

The results of MCC tests are shown in Figure 7 and Table 8 and Table 9. All samples underwent combustion in two stages. The first peak occurred in the range of 595–665 K, and the second peak in the range of 702–730 K. In the first stage (at lower temperatures), the polylactide phase decomposed first, followed by combustion of the PET phase in the second stage. In samples 1:2, 1:2/CLS, 1:2/SA, 1:2/BMT, 1:1, 1:1/CLS, 1:1/SA, 2:1/SA, the dominant combustion stage (i.e., the stage with the highest heat release) was the second. Conversely, in samples 1:2/BMG, 1:1/BMT, 1:1/BMG, 2:1, 2:1/BMT, and 2:1/BMG the first stage was dominant. It is worth noting that the nature of the individual combustion stages was influenced by the proportion of the appropriate additive in the composite blend system. For example, as the PLA content in the blend increased, the effectiveness of CLS in reducing its flammability diminished, favoring the PET subphase instead. Thus, in the 2:1/CLS sample, the flammability of the dominant phase decreased slightly (only 4%) compared to the 2:1 sample, while that of the PET phase decreased by 65%. In contrast, the addition of SA, in all the cases considered, inhibited only the combustion of the PLA phase, and this effect was stronger with higher PLA content. Notably, a 76% reduction in the PHRR_PLA_ value was achieved for the 2:1/SA sample compared to the reference sample (2:1). The presence of BMT had a noticeable effect on the flammability of the PET phase, particularly in the 1:1/BMT sample. A 48% reduction in the PHRR_PET_ value was achieved compared to the 1:1 sample (see Table 8). Meanwhile, BMG inhibited only the combustion of the PET phase. This inhibition was more pronounced when the PET content was lower. For the 2:1/BMG sample, a 32% reduction in PLA flammability and a 76% reduction in PET flammability were achieved compared to 2:1. All biofillers also caused shifts in the PHRRₚₗₐ peaks toward lower temperatures, averaging around 40 K. In contrast, the effect on PHRRₚₑₜ peak positions was negligible, with an average shift in only ~6 K.

All biofillers caused an average acceleration of ignition in composite blends, with CLS reducing ignition time by 65 s, SA by 41 s, BMT by 61 s, and BMG by 93 s. Most of the used additives also promoted faster Time-Out-Flame (TOF). Only two exceptions were noted: 1:2/CLS and 1:1/SA, in which the TOF values were similar to the reference samples. Importantly, regardless of the share of individual polymer phases in the blend, the use of each type of bioadditive led to an extension of the combustion time. Among the blends with dominant PET phase, the samples containing CLS and BMG burned the longest. Among the blends with equal proportions of both polymer phases, the 1:1/SA and 1:1/BMG samples burned the longest. For the samples where the PLA phase dominated, the 2:1/BMG and 2:1/BMT samples burned the longest. It is worth noting that as the PLA phase content increased share in the blends, the combustion time of samples containing CLS gradually decreased, whereas that of samples containing BMG gradually increased.

Table 9 presents additional flammability indices calculated according to the ASTM D7309 standard, which is intended for analyzing results obtained from the MCC test. By analyzing only the reference samples, it was observed that the heat release capacity (η_c_) value increased with the increase in the proportion of the PLA phase in the blends. This result was not surprising, as PLA is a biopolymer much more susceptible to ignition than PET. This is primarily due to PLA’s lower thermal stability and lower char-forming ability, which leads to faster decomposition and greater release of flammable volatiles compared to PET [21]. Therefore, it was expected that increasing its mass fraction in the blend system would increase the blend’s heat-releasing capacity. This trend did not change even after the addition of fillers such as BMT and BMG. Furthermore, in composite blends containing BMT, the fire growth capacity (FGC) values also increased with increasing biopolymer phase content. An interesting but opposite effect was observed for composite blends containing CLS and SA. That is, in samples containing CLS, the FGC values increased with increasing PLA phase, whereas in samples containing SA, the flame propagation ability decreased with increasing biopolymer phase. The increase in FGC with CLS could be related to its role in catalyzing the thermal degradation of the biopolymer phase, potentially enhancing volatile production [98]. Conversely, SA may promote char formation or act as a radical scavenger, thereby inhibiting flame propagation. This indicates that pure calcium lignosulfonate enhanced the combustion effect of the biopolymer phase, while lignosulfonamide acted as an inhibitor. In 1:2 and 1:1 composite blends, the highest FGC values were recorded for samples containing SA. Among the 2:1 blends, the highest flame spread ability was observed for 2:1/CLS. Consistent with this, the lowest FGC values were observed for samples containing BMG, regardless of the polymer phase content. In 1:2 and 2:1 composite blends, the highest and lowest η_c_ values were recorded for samples containing CLS and BMG, respectively. In 1:1 blends, the effect was reversed, i.e., the highest eta values were observed for the 1:1/BMG sample and the lowest for the 1:1/CLS sample, which confirms the results presented in Table 8. It is worth adding that the lowest or much lower values of the total heat release rate (THR, h_c_) compared to the reference sample were found in blends containing gallic acid (BMG), which makes this additive relatively the most effective for this type of polyester blends. Gallic acid’s effectiveness may be attributed to its antioxidant properties, promoting char formation and scavenging flame-propagating radicals, which slows down thermal degradation and heat release [99].

**Figure 8:**
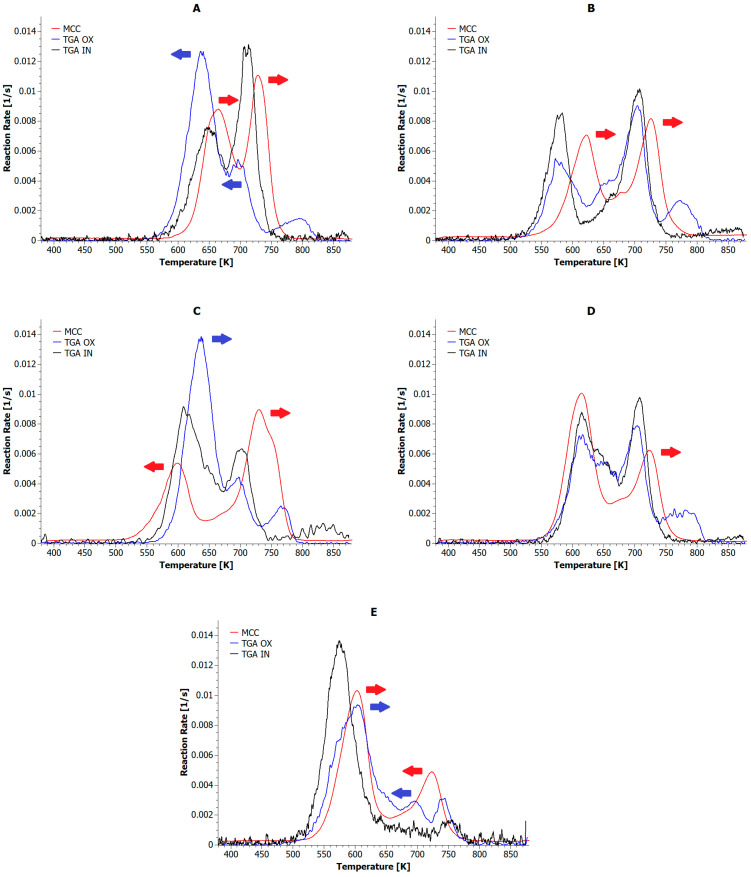
Decomposition Reaction Rates of PLA+PET blends performed in MCC and TGA (an oxidizing and inert atmosphere) tests: (**A**) pure blend; (**B**) including CLS; (**C**) including SA; (**D**) including BMT; (**E**) including BMG.

The comparison of the decomposition rate curves for the composite blends confirms previous report [13,22,47,48] of a multi-stage decomposition process under oxidizing conditions. Figure 8 compares the decomposition rates of 1:1 PLA:PET blend samples during MCC and TGA under both oxidizing and neutral conditions.

The 1:1 pyrolyzed sample showed two decomposition reaction rate (RR) maxima, at 650 and 712 K. This indicates that the decomposition of the individual phases was most effective at these temperatures, corresponding to peak release of volatile products. Rapid combustion of these samples shifted both of these maxima by approximately 15 K toward higher temperatures, while decomposition under oxidizing conditions shifted both of these maxima by approximately 15 K toward lower temperatures. During thermo-oxidative decomposition, a third band appeared with a maximum at 796 K, and the first band showed the highest reaction rate. It can be concluded that the decomposition rate depended directly on the conditions under which it was carried out, the reference samples decomposed fastest under oxidizing conditions and slowest during controlled combustion. The introduction of CLS stabilized the slow decomposition process. Regardless of the prevailing process conditions, very similar results were observed, with the PET phase decomposing the fastest. In turn, the fastest combustion points of the PLA and PET phases shifted to the right by 42 K and 21 K, respectively, relative to the fastest pyrolysis point. Looking at the RR curves of the composite blends containing SA, one can conclude that the decomposition rate of these samples indicated a highly irregular decomposition profile, suggesting the absence of a defined degradation pathway under all measurement conditions. No specific trend can be discerned in this case, which may limit the utility of lignosulfonamide for recovering valuable degradation products. However, blends containing SA certainly exhibited an extended decomposition temperature range in the presence of oxygen. The most promising results are presented in Figure 8E. They indicate that BMT can exhibit strong catalytic activity in decomposition processes under all environmental conditions. In each case, low RR curve peak values were obtained, not exceeding 0.01 1/s. Thus, BMT caused equal decomposition rates for both polymer phases, especially when the temperature increase was slow. The use of BMG aims to achieve a single, coordinated decomposition reaction between two immiscible polyester phases. This may be due to BMG selectively inhibiting or catalyzing the decomposition of the PET phase, whose decomposition rate disappears under anaerobic conditions and gradually decreases under aerobic conditions.

To quantitatively assess the effect of bio-additives on the thermal stability and flammability of composite blends, the Overall Thermal Stabilization Effect (*OSE*) and Overall Flame Retardancy Effect (*OFRE*) values were determined using the following Equations (2) and (3) [100,101,102,103]:

(2) $$\begin{array}{cc} OSE=\sum_{T=423}^{873} & \left(({mass\;percent\;of\;composite\;blend}_{T}\right)\\ & \;-({mass\;percent\;of\;pure\;polymer\;blend}_{T})) \end{array}$$

(3) $$\begin{array}{cc} OFRE=\sum_{T=423}^{873} & \left(({HRR\;percent\;of\;pure\;polymer\;blend}_{T}\right)\\ & \;-({HRR\;percent\;of\;composite\;blend}_{T})) \end{array}$$

**Figure 9:**
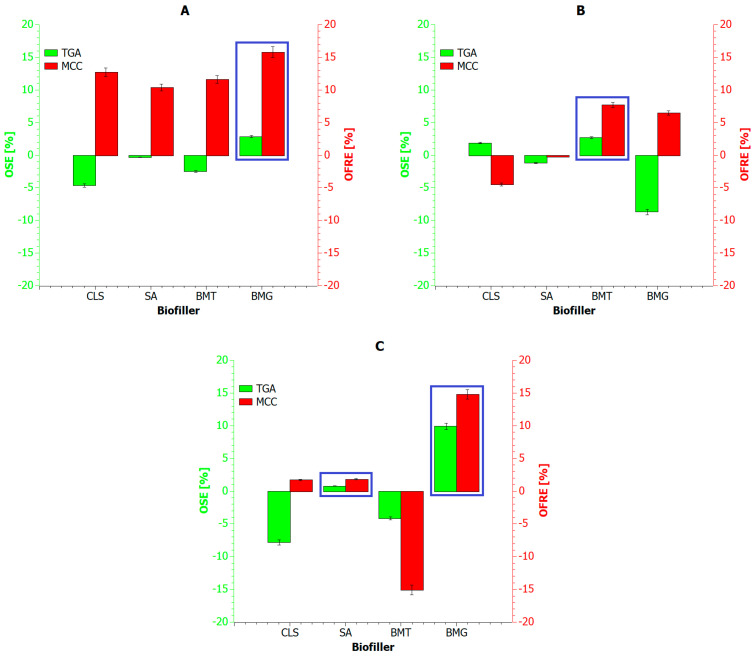
Overall Thermal Stabilization Effect (*OSE*) and Overall Flame Retardancy Effect (*OFRE*) for all tested composite blends: (**A**) the PLA:PET phases in ratio of 1:2; (**B**) the PLA:PET phases in ratio of 1:1; (**C**) the PLA:PET phases in ratio of 2:1.

The *OSE* and *OFRE* values obtained for the composite blends thus relate directly to the reference samples (Figure 9). Therefore, when comparing the CLS samples, a certain pattern was observed: the dominance of one of the two blend phases fraction led to reduced thermal stability while simultaneously improving fire resistance. However, this resistance increased with the proportion of the phase having a higher glass transition temperature (T_g_) and melting point (T_m_). This indicates that CLS increased the susceptibility of the biopolymer phase to decomposition, which resulted in the formation of highly volatile gaseous products capable of cooling the remaining PET phase in the system and initiating the formation of a surface char layer earlier.

In turn, didodecyl-lignosulfonamide had a minimal effect on the overall thermal stability of the composite blends. *OSE* values were very similar to the reference samples, and positive *OSE* and *OFRE* values were obtained only for the 2:1/SA sample. The above analysis confirms the low or selective reactivity of this additive under oxidizing conditions, suggesting that an additional factor may be required to activate its action at elevated temperatures.

The use of an additive rich in tannic acid provided good thermal stabilization and fire resistance only when the ratio of both polymer phases was balanced. This suggests that tannic acid provides more effective flame protection when the biopolymer phase is reduced or less dominant in the blend. This is due to the very rapid decomposition of CLS and TA, leading to the emission of non-flammable gases. When the PET phase dominates the system, these gases play a protective and cooling role. However, when the PLA phase dominates, the rapid release of acidic gases (CO, CO_2_, and SO_2_) eliminates the protective barrier that would otherwise inhibit polyester decomposition.

The most effective additive was the BMG component. Regardless of the PLA:PET mass ratio, BMG significantly improved the flame resistance (*OFRE*) compared to the reference samples. An advantage of using gallic acid is that its thermal decomposition releases compounds (primarily organic acids) that are considered good flame retardants. Therefore, both polymer phases are constantly surrounded by decomposition products that scavenge peroxide radicals and promote char formation.

The UL-94 HB (horizontal burning) test results are presented in Table 10, and the UL-94 VB (vertical burning) test results are presented in Table 11. All samples subjected to flammability testing burned completely. For the reference PLA+PET samples (1:2, 1:1, 2:1), the average combustion time was 92, 98 and 105 s, respectively. Furthermore, the change in combustion rate was analyzed by comparing the combustion time of the first and second half of the sample. It was observed that the combustion rate of the second measurement section was slower for all tested composite blends. Additionally, a thin layer of charring generally appeared on the edges of the blend samples dominated by the biocomposite phase (2:1).

**Figure 10:**
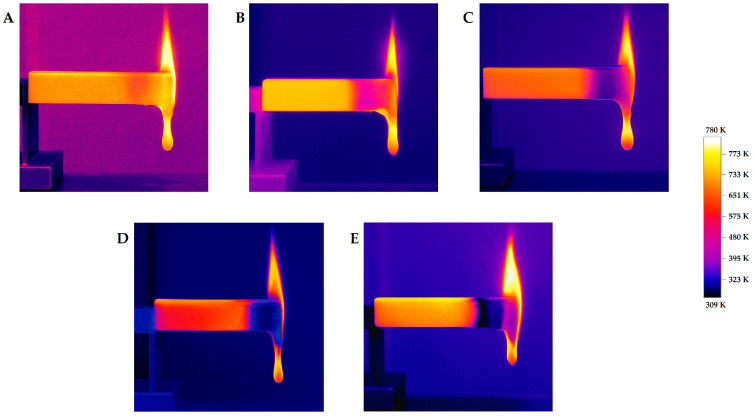
Thermal photos of selected samples burned in the UL94 HB test: (**A**) 1:1 blend; (**B**) 1:1/CLS composite blend; (**C**) 1:1/SA composite blend; (**D**) 1:1/BMT composite blend; (**E**) 1:1/BMG composite blend.

Composite blends containing BMG achieved the longest average burn times compared to the reference samples. The overall slowdown in combustion was due to BMG’s effect on the flame spread rate (FGC), which was confirmed by previous analysis. Regardless of the type of additive used, char formation was observed during combustion of the composite blends (Figure 10). Based on these observations, a clear relationship was observed: as the amount of char formation increased, the number of droplets detaching from the burning sample decreased. In the reference samples and those containing CLS and SA, the number of falling droplets increased with increasing PLA phase content. This trend was disrupted by the use of BMT additives, and especially BMG.

Table 11 presents the results of the UL-94 VB test. The main criteria for assigning the tested composite blends to a specific flammability class were their flammability properties, defined as the material’s combustion time and the behavior of flaming or non-flaming dripping fragments, as defined by the UL-94 standard. In this case, classes FV-1 and FV-2 were distinguished. Class FV-1 refers to materials whose molten polymer fragments do not ignite the cotton placed beneath the sample, and where the flame self-extinguishes without propagating significantly. Class FV-2 refers to materials whose molten alloy still does not ignite the cotton placed under the sample, which exhibit longer burning times or flame propagation compared to FV-1. Accordingly, class FV-1 included all blends containing BMT and BMG, and class FV-2 included all blends containing SA. Blends containing CLS and reference samples did not meet the requirements of the standard and were not assigned to any flammability class.

As expected, the cotton ignited after a large number of droplets had fallen (CLS and SA composite blends, and reference samples). Furthermore, the vertical orientation of the sample favored rapid flame movement from the bottom up. Virtually all of the reference samples heated under these conditions melted rapidly and flowed with the flame onto the cotton fabric placed underneath. The rapid temperature rise reduced the melt viscosity so much that, without additional factors, the sample geometry was not stable. Only the presence of lignosulfonamide and CLS modified with tannic and gallic acids in the system slowed the combustion process, prolonging the effect of mutual cohesion. Generally, as the number of falling droplets decreased, the combustion time of the samples increased. Observations indicate that in samples containing SA, BMT, and BMG, a layer of char mixed with the melt rapidly formed on the surface, effectively enclosing the sample core within the developing pseudo-scaffold. This resulted in only surface combustion/charring of the material, followed by melting and degradation of the material on the inside. The extent of the processes occurring in the core of the burning material varied between cases.

**Figure 11:**
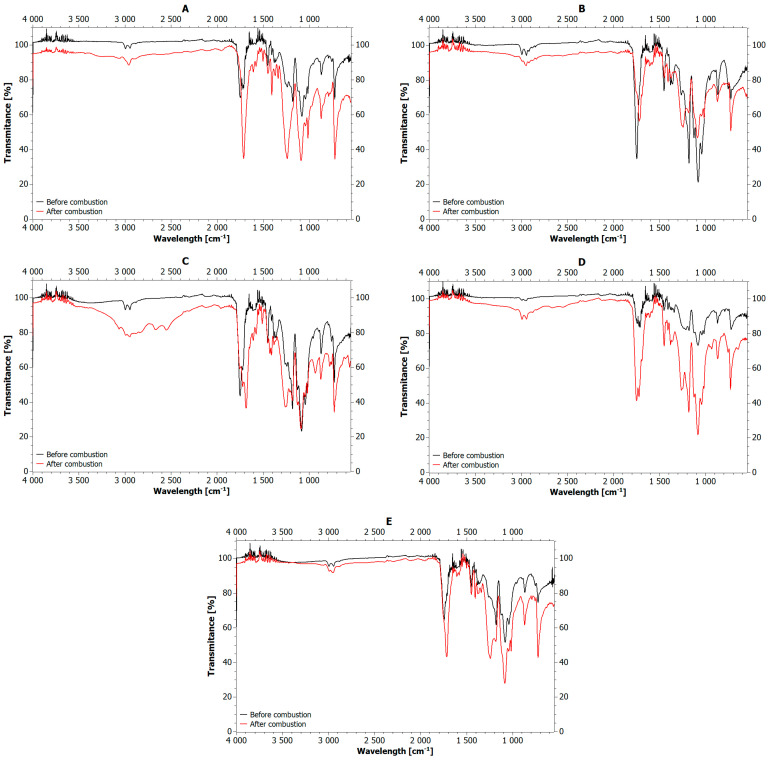
Comparison of FTIR spectra of 1:1 composite blends before and after combustion: (**A**) pure PLA+PET blend; (**B**) including CLS; (**C**) including SA; (**D**) including BMT; (**E**) including BMG.

To understand this mechanism, the char remaining after combustion of the samples was analyzed for the presence of functional groups and compared with the FTIR spectra of these samples before combustion. The result of this comparison is shown in Figure 11.

In all considered cases, both before and after combustion, an absorption band in the range of 3032–2860 cm^−1^ was present, originating mainly from C-H stretching vibrations in the aliphatic chain (with possible contribution from aromatic C–H near ~3030 cm^−1^) [104,105]. However, in the char samples, it was much more intense. Perhaps initial cracking of polymer chains initiated during processing directed the decomposition of these compounds along similar pathways. In both the blend and char samples, some common absorption bands were noted, i.e., in the range of 1751–1712 cm^−1^ from C=O stretching vibrations, 1250–1239 cm^−1^ from C–O stretching vibrations, around 1087 cm^−1^ a band from C-O stretching vibrations and bands at 871 cm^−1^ and 725 cm^−1^ from C-H bending vibrations in isomers of aromatic compounds [106,107,108,109]. Typically, the intensity of these absorption bands was higher in the samples after combustion, suggesting the formation of carboxylic acids and their aromatic ester derivatives. For example, in the pure blend sample, characteristic bands were observed at 1452 and 1179 cm^−1^, from CH_2_ bending/scissoring and C-O stretching vibrations, respectively. In the residue of this sample, additional bands were found at 1504 cm^−1^, and 1407 cm^−1^, which are consistent with aromatic C=C and ring modes [110].

The addition of CLS biofiller to the blend resulted in additional bands, also visible after combustion, in the ranges of 1605–1580 cm^−1^ and 1452 cm^−1^, from stretching and deformation vibrations of carbonyl and hydroxyl groups, respectively. The most pronounced changes occurred in blends containing lignosulfonamide (SA). In these samples, the presence of nitrogen was observed at 2129 cm^−1^ prior to combustion. However, after combustion, the following functional groups were visible: OH—3066 cm^−1^, SH—2665 and 2550 cm^−1^, C≡C—1948 cm^−1^; C≡N—1682 cm^−1^; C=C—1511 cm^−1^, and C-H—933 cm^−1^. Sulfur-containing bonds were also activated during the combustion of BMT and BMG samples, as indicated by new intense bands at 1408 cm^−1^, 1308 cm^−1^, suggesting the formation of various hydroxy dicarboxylic acids enantiomers, including tartaric acid.

The above analysis confirms that the flame-retardant mechanism of SA is primarily based on the cleavage of the nitrogen-sulfur core present in lignosulfonamide. The residual amide group decomposes, releasing nitrogen gas. Oxidation of the sulfonate group leads to the release of SO_2_, which facilitates char formation. Tannic and gallic acids, in turn, exhibit significant synergy with the sulfonic groups present in CLS. Decomposition of gallic acid produces a complex mixture of hydroxycarboxylic acids, including vanillic acid, phthalic acid, benzoic acid, ellagic acid, and tannic acid, with tartaric acid being the predominant product. Further heating in the presence of sulfonic groups produces aromatic hydrocarbons, phenolic compounds, SO_2_, CO_2_, and CO, and ultimately leading to char formation.

The limiting oxygen index (LOI) is defined as the minimum oxygen concentration in an oxygen-air mixture required to sustain combustion of the tested material. LOI measurements began with reference samples, for which 21% was selected as the baseline. Table 12 presents the LOI values obtained for the analyzed blends.

The measurements showed that none of the analyzed blends ignited under oxygen-deficient conditions. This means that the oxygen concentration in the air must be increased to ignite even the reference samples. This is primarily due to the presence of the PET phase, which is a polyester that is difficult to ignite under standard conditions. The higher the PET content in the blend, the more difficult it was for the test material to ignite. All additive types increased the LOI values relative to the reference samples. The lowest changes were observed for CLS, although this additive is believed to be the most compatible with the PLA phase. As the biopolymer content in the system increased, the ∆LOI of the CLS samples also gradually increased. As expected, the samples containing BMT and BMG were the most difficult to ignite. Although the BMT filler performed poorly in the 1:1 system, ignition of either of these samples typically required an additional 3–4% oxygen compared to the reference.

### 5.3. Potential Upcycling and Recycling Paths

PLA+PET blends modified with bio-based additives such as CLS, SA, BMT, and BMG exhibit complex thermal and combustion properties. The obtained thermal, structural, and flammability data open up diverse possibilities for further processing, both in the context of material/energy recycling, as well as upcycling. A well-designed management strategy for such polymer waste can significantly reduce its environmental impact, improve the product’s life economics, and enable new applications with higher utility value.

Data show that additives such as CLS and BMG increase the degree of crystallinity, particularly in 1:1 and 2:1 systems. High crystallinity improves stiffness, barrier properties, and fire resistance. These materials can be upcycled for semi-technical or consumer applications, such as components for electronics housings, reusable packaging, technical parts in automotive, and household appliances, where thermal resistance and dimensional stability are desirable. The pronounced T_g_ shifts and crystallization behavior suggest the possibility of controlled foaming of these mixtures using nucleating additives. The ability to create technical foams with controlled microstructure (particularly from 1:1/CLS, 2:1/BMG mixtures) may lead to the creation of lightweight, rigid thermal or sound-absorbing materials.

High thermal stability (especially in the presence of SA and BMG), controlled flammability, and consistent physical properties make selected blends suitable for processing into 3D printing filaments. In this case, blends with a 2:1/BMG and 1:1/SA composition are of interest, as they demonstrate favorable flammability parameters, long combustion times, and the ability to form a protective char layer.

PLA+PET blends containing BMG and BMT, classified as FV-1, can be used as flame-retardant cable sheaths, technical components for the transportation industry (e.g., train or bus interiors), and in the construction sector (e.g., ceiling cassettes, wall panels). A promising combination is PLA:PET = 1:1 with BMT, which offers relatively high thermal stability along with low flammability.

The apparent immiscibility of the PLA and PET phases (evidenced by the presence of two distinct T_g_ and T_m_ points) suggests that, under suitable processing conditions, physical separation of the polymers is feasible at the end of the product life cycle. Mechanical or chemical recycling can be performed separately for the PLA phase (e.g., re-hydrolysis to lactic acid) and the PET phase (e.g., glycolysis to terephthalate monomers).

The high thermal reactivity of some additives (e.g., BMG, SA) can be utilized in catalytic processes, for example, to initiate the depolymerization of PLA or the decomposition of PET into its basic components. BMG, containing carboxyl groups, can act as a mild catalyst in acidic environments, supporting the recovery of ester compounds. Unfortunately, according to the specialist literature [111,112,113,114], thermo-oxidative and thermal decomposition of polyesters is such a complicated process that the selective recovery of polyester monomers using this method becomes almost impossible to implement. The random scission mechanism of the PLA backbone results in the formation of other degradation products, such as CO, CO_2_, acetaldehyde and methyl ketone, in addition to lactic acid and lactide oligomers [115,116]. On the other hand, the main products of thermo-oxidative degradation of PET are cyclic and linear (di-acidic end groups) oligomers [117,118]. During the pyrolysis of PET, both volatile degradation products and non-volatile residues are formed. The production of volatile substances occurs through two degradation routes: one leading to cyclic oligomers and the other involving hydrogen transfer, which causes monomer loss [119,120]. Degradation kinetics in 623–643 K range also confirms the formation of residues rich in aromatic hydrocarbons [110].

Under anaerobic conditions (TGA in an inert atmosphere), these blends exhibited significant thermal stability and left substantial residues (up to 10.5%). These residues can potentially be converted into technical carbon, black pigment, or adsorbent (e.g., for gas purification). Blends containing BMT and SA are particularly suitable for this purpose.

PLA+PET blends exhibit a two-phase combustion profile, enabling controlled heat release rates and improved energy management. Due to the different peak heat release temperatures for the PLA phase (approximately 600–660 K) and PET (700–730 K), the combustion process can be modulated more gradually. These materials could serve as supplement fuels in waste-to-energy incineration plants, especially blends with the lowest emissions (e.g., those containing BMG).

The high content of aromatic groups and esters in combustion residues suggests their potential utility for further organic synthesis or as precursors for alternative fuel production. Concurrently, blends with BMG additive provided the lowest total heat release (THR) values, suggesting their safer energy use in closed systems.

Systematic analysis of DSC, TGA, and flammability profiles enables the classification and selection of suitable processing pathways, based on mixture composition and bioactive additives used. The final decision between recycling and upcycling should consider the available technological infrastructure, energy value, and market demand for materials with enhanced thermal or flammability resistance.

## 6. Conclusions

The addition of biofillers such as calcium lignosulfonate (CLS), lignosulfonamide (SA), tannic acid-modified lignosulfonate (BMT), and gallic acid-modified lignosulfonate (BMG) significantly alters the thermal, crystalline, and flammability properties of PLA+PET blends. Their effect depends on both the nature of the additive and the PLA-to-PET weight ratio.

DSC confirmed the partial immiscibility of PLA and PET in the blend, indicated by the presence of two distinct glass transitions and two melting peaks. Some additives (e.g., BMT) modified the mobility of the chain segments, thereby altering the thermal transition behavior.

TGA showed that the additives could both lower the degradation onset temperature (BMG) and increase the char yield (BMT, SA), suggesting distinct thermal degradation mechanisms for each additive under oxidative conditions.

The most fire-resistant properties were achieved in blends containing BMG, which exhibited a reduced combustion rate, prolonged ignition delay, and increased char formation were observed. BMT also improved thermal stability and flame resistance, although to a lesser extent. Only the BMG and BMT samples achieved a flammability classification of FV-1, while SA was rated FV-2. The reference and CLS containing samples did not meet the minimum flammability classification criteria. BMT influence on the flammability of polyester blends could be explained by its ability to promote char formation and inhibit volatile release during thermal decomposition. This results in the formation of a protective barrier on the surface of the material, limiting oxygen access and heat transfer, thereby reducing the overall combustibility.

Among all tested additives, BMG demonstrated the highest potential as a bio-based flame retardant for PLA+PET blends, offering both high flame retardancy and environmental compatibility. It has been postulated that the BMG mode-of-action involves the release of non-flammable gases and the formation of a stable aromatic char layer during degradation. The presence of gallic acid moieties may enhance crosslinking and carbonization, effectively insulating the underlying material and slowing down the combustion process.

In general, the incorporation of such biofillers represents a promising strategy for designing safer and more sustainable polymer composites that meet the current technical and environmental requirements.

## Figures and Tables

**Table 1:** Processing conditions.

Extrusion							
**Temperature [K]**	**Heating Zones**						
	**1**	**2**	**3**	**4**	**5**	**6**	**Die**
	493	498	503	508	513	513	513
**Degassing**	-	-	-	-	Yes	-	-
**Screws Speed [rpm]**	150						
**Feed Capacity [%]**	5						
**Cooling Bath**							
**Temperature [K]**	293						
**Compression Press**							
**Temperature of Stamps [K]**	513						
**Pressure [Bar]**	240						
**Pressing Time [s]**	420						

**Table 2:** Survey of the obtained composite blends.

Sample No.	PLA: PET Mass Ratio	Biofiller				Sample Designation
		CLS	SA	BMT	BMG	
1	1:2	-	-	-	-	1:2
2		✓	-	-	-	1:2/CLS
3		-	✓	-	-	1:2/SA
4		-	-	✓	-	1:2/BMT
5		-	-	-	✓	1:2/BMG
6	1:1	-	-	-	-	1:1
7		✓	-	-	-	1:1/CLS
8		-	✓	-	-	1:1/SA
9		-	-	✓	-	1:1/BMT
10		-	-	-	✓	1:1/BMG
11	2:1	-	-	-	-	2:1
12		✓	-	-	-	2:1/CLS
13		-	✓	-	-	2:1/SA
14		-	-	✓	-	2:1/BMT
15		-	-	-	✓	2:1/BMG

**Table 3:** The biofillers’ TGA indices.

Sample	T_5%_ [K]	T_10%_ [K]	T_20%_ [K]	T_50%_ [K]	T_MAX_ [K]	Residue at 873 K [%]
CLS	361	449	527	667	723	13.10
SA	384	465	548	---	746	66.60
BMT	367	457	527	666	703	14.12
BMG	386	428	471	628	504	39.06

**Table 4:** The biofillers’ combustion indices determined based on flammability curves, where PHRR—peak of heat released rate; HRR_AV_—average heat released rate; HOC_AV_—average heat of combustion; THR_600s_—total heat released after 600 s; TTI—time to ignition; TOF—time out flame.

Sample	PHRR [W·g^−1^]	HRR_AV_ [W·g^−1^]	HOC_AV_ [kJ·g^−1^]	THR_600s_ [kJ·g^−1^]	TTI		TOF		Combustion Time [s]
					[s]	[K]	[s]	[K]	
CLS	23	12	3.86	7.26	120	448	384	712	319
SA	35	8	2.73	4.60	115	423	558	863	443
BMT	26	12	3.81	6.85	154	468	425	742	271
BMG	39	11	4.13	6.31	3	382	411	727	408

**Table 6:** TGA indicators of composite blends Where OX—under oxygen conditions; IN—test under inert atmosphere.

Sample	T_5%_ [K]		T_10%_ [K]		T_20%_ [K]		T_50%_ [K]		T_MAX_ [K]		Residue at 873 K [%]	
	OX	IN	OX	IN	OX	IN	OX	IN	OX	IN	OX	IN
1:2	606	598	620	619	635	638	673	688	701	711	0.00	5.18
1:2/CLS	543	554	554	570	569	591	678	694	705	710	1.54	9.97
1:2/SA	602	583	614	595	630	616	675	692	704	711	0.51	9.99
1:2/BMT	573	589	587	608	607	656	671	701	701	710	0.95	10.51
1:2/BMG	575	555	597	573	645	594	696	676	707	699	0.04	0.00
1:1	596	588	609	611	622	629	648	661	637	657	0.00	0.00
1:1/CLS	555	541	569	558	589	575	676	673	704	707	1.51	4.89
1:1/SA	598	512	608	585	620	602	644	636	636	609	0.00	0.00
1:1/BMT	580	587	597	600	613	614	666	664	703	709	0.60	6.86
1:1/BMG	543	527	558	543	575	557	611	581	604	576	1.45	0.00
2:1	593	571	604	587	617	603	638	642	636	613	0.00	0.56
2:1/CLS	552	536	565	555	580	569	608	591	611	588	1.04	0.00
2:1/SA	595	568	607	586	618	603	639	641	634	614	0.00	0.44
2:1/BMT	581	568	597	586	610	603	629	655	632	609	0.46	2.57
2:1/BMG	575	556	595	569	622	583	687	658	704	711	0.35	0.00

**Table 7:** Comparison of TGA results obtained under oxidizing (OX) and inert (IN) conditions.

Feature	OX Conditions	IN Conditions
T_MAX_ range	604–707 K	576–711 K
Best sample up to T_20%_ point	1:2	1:2
Worst sample up to T_20%_ point	1:2/CLS	1:1/BMG
Best sample above T_20%_ point	1:2/BMG	1:2/BMT
Worst sample above T_20%_ point	1:1/BMG	1:1/BMG
Char residue	Very low (0–1.5%)	High (up to 10.5%)
Additive improving stability	---	BMT, SA
Additive decreasing stability	CLS, BMG	CLS, BMG
Degradation character	Combustion (full degradation)	Pyrolysis, carbonization

**Table 8:** The PLA+PET composite blends’ combustion indices determined based on flammability curves. Where PHRR —peak of heat released rate; HRR_AV_—average heat released rate; HOC_AV_—average heat of combustion; THR_600s_—total heat released after 600 s; TTI—time to ignition; TOF—time out flame.

Sample	PHRR_MAX_ [W·g^−1^]	PHRR_PLA_ [W·g^−1^]	PHRR_PET_ [W·g^−1^]	HRR_AV_ [W·g^−1^]	HOC_AV_ [kJ·g^−1^]	THR_600s_ [kJ·g^−1^]	TTI		TOF		Combustion Time [s]
							[s]	[K]	[s]	[K]	
1:2	242	148 (645 K)	242 (727 K)	32.81	10.59	22.39	263	574	479	796	216
1:2/CLS	272	91 (595 K)	272 (721 K)	28.87	9.44	19.74	196	509	478	797	282
1:2/SA	244	109 (623 K)	244 (724 K)	29.76	9.81	20.43	236	559	466	794	230
1:2/BMT	182	132 (619 K)	182 (724 K)	29.38	10.07	20.11	213	538	468	798	255
1:2/BMG	157	157 (608 K)	135 (722 K)	28.41	9.99	19.03	202	513	474	792	272
1:1	240	191 (665 K)	240 (728 K)	30.25	9.96	21.56	264	568	487	797	223
1:1/CLS	185	160 (623 K)	185 (726 K)	32.22	11.02	22.00	200	517	477	802	277
1:1/SA	195	117 (599 K)	195 (730 K)	30.57	10.33	21.45	197	503	490	803	293
1:1/BMT	201	201 (615 K)	125 (723 K)	28.76	10.51	19.91	214	536	474	803	260
1:1/BMG	209	209 (603 K)	99 (723 K)	29.68	11.00	19.96	174	503	463	798	289
2:1	353	353 (654 K)	94 (726 K)	31.24	10.83	21.86	267	573	475	787	208
2:1/CLS	340	340 (634 K)	33 (711 K)	31.02	11.61	21.43	203	514	456	773	253
2:1/SA	300	83 (633 K)	300 (728 K)	31.20	9.88	21.32	239	564	467	797	228
2:1/BMT	295	295 (630 K)	65 (715 K)	36.80	12.93	24.34	183	508	445	776	262
2:1/BMG	241	241 (607 K)	23 (702 K)	26.85	10.46	18.29	139	455	459	776	320

**Table 9:** Flammability factors for polylactide composites calculated according to ASTM D7309 standard, where FGC—fire growth capacity of sample; h_c_—specific heat release in the test; η_c_—heat release capacity; T_5%_—temperature in the test at which 5% of h_c_ has been released measured at a heating rate β = 1 K/s; T_95%_—temperature in the test at 95% of h_c_ has been released measured at a heating rate, β = 1 K/s.

Sample	T_5%_		T_95%_		FGC [J/gK]	η_c_ [J/gK]	h_c_ [kJ/g]
	[s]	[K]	[s]	[K]			
1:2	219	529	555	873	167.53	269.08	23.15
1:2/CLS	233	546	517	836	151.14	302.61	20.21
1:2/SA	264	588	396	723	225.19	266.94	20.74
1:2/BMT	236	560	486	816	158.10	199.22	20.47
1:2/BMG	216	544	525	860	140.94	170.18	19.49
1:1	312	618	442	752	229.25	271.33	21.65
1:1/CLS	236	554	541	865	161.20	204.63	22.64
1:1/SA	240	547	455	767	185.92	219.76	21.72
1:1/BMT	244	566	421	748	184.40	220.93	19.99
1:1/BMG	210	539	459	793	163.65	227.43	20.24
2:1	290	596	439	750	217.86	395.58	22.12
2:1/CLS	246	558	444	760	191.33	380.82	21.75
2:1/SA	249	574	493	823	165.86	330.27	21.71
2:1/BMT	215	540	587	918	172.54	323.05	25.46
2:1/BMG	209	521	540	858	140.36	268.21	18.84

**Table 10:** Measurement results using the UL-94 HB flammability technique.

Sample	Average Combustion Time [s]	Average Combustion Rate [mm/min]		Length of the Burned Section [mm]	Average Number of Drops
		1 *	2 *		
1:2	92	61.1	48.9	75	64
1:2/CLS	58	97.0	77.6		38
1:2/SA	78	72.1	57.7		25
1:2/BMT	111	50.7	40.5		19
1:2/BMG	114	49.3	39.5		18
1:1	98	60.2	45.9	75	82
1:1/CLS	62	95.1	72.6		49
1:1/SA	86	68.5	52.3		27
1:1/BMT	105	56.1	42.9		20
1:1/BMG	114	51.7	39.5		15
2:1	105	57.0	42.9	75	96
2:1/CLS	63	95.0	71.4		54
2:1/SA	90	66.5	50.0		32
2:1/BMT	98	61.1	45.9		25
2:1/BMG	118	50.7	38.1		14

* Burning rate for the first (1) and second (2) long sections

**Table 11:** Measurement results using the UL-94 VB flammability technique.

Sample	Average Combustion Time [s]	Fabric Inflammation	Flame Progressed Up to the Holding Clamp	Average Number of Drops	Type of Standard Class
1:2	40	YES	YES	20	-
1:2/CLS	47	YES	YES	11	-
1:2/SA	57	YES	NO	3	FV-2
1:2/BMT	64	NO	NO	4	FV-1
1:2/BMG	78	NO	NO	3	FV-1
1:1	36	YES	YES	31	-
1:1/CLS	45	YES	YES	12	-
1:1/SA	52	YES	NO	5	FV-2
1:1/BMT	55	NO	NO	5	FV-1
1:1/BMG	77	NO	NO	4	FV-1
2:1	30	YES	YES	37	-
2:1/CLS	42	YES	YES	12	-
2:1/SA	48	YES	NO	10	FV-2
2:1/BMT	53	NO	NO	6	FV-1
2:1/BMG	68	NO	NO	4	FV-1

**Table 12:** LOI value summary for PLA+PET composite blends.

Sample	LOI [% Oxygen]	∆LOI [% Oxygen]
1:2	22.8 ± 0.1	---
1:2/CLS	23.5 ± 0.1	0.7
1:2/SA	25.5 ± 0.1	2.7
1:2/BMT	26.4 ± 0.1	3.6
1:2/BMG	27.5 ± 0.1	4.7
1:1	22.0 ± 0.1	---
1:1/CLS	23.4 ± 0.1	1.4
1:1/SA	24.1 ± 0.1	2.1
1:1/BMT	24.8 ± 0.1	2.8
1:1/BMG	27.1 ± 0.1	5.1
2:1	21.0 ± 0.1	---
2:1/CLS	23.1 ± 0.1	2.1
2:1/SA	24.4 ± 0.1	3.4
2:1/BMT	24.8 ± 0.1	3.8
2:1/BMG	24.9 ± 0.1	3.9

## Data Availability

The original contributions presented in this study are included in the article material. Further inquiries can be directed to the corresponding authors.
